# Notes and News

**Published:** 1897-10-30

**Authors:** 


### Notes and News.

(Collected and Communicated.)

The deaths from yellow fever at New Orleans have exceeded 100. The number of persons attacked with the disease is upwards of 950.

A new infectious hospital has been opened near Motherwell, Lanarkshire. It will accommodate 100 patients.

A large naval hospital is to be built on Haulbowline Island, near Queenstown, Ireland. It is intended to utilise the Haulbowline Docks and workshops for the repair of naval ships, and to place the hospital on a quiet part of the island as far from the works as possible.

Mr. Passmore Edwards has promised to erect a convalescent home to accommodate 50 patients, and costing £6,000, for the use of members of the principal registered friendly societies in the country. A site has been secured about a mile from Herne Bay, comprising seven acres.

Dr. M'Kelvie has presented Oban with a very complete small isolation hospital at a cost of £2,000. It is built on the pavilion principle, and consists of four wards, an administration block, and a disinfecting department, and is equipped with approved modern appliances. The hospital was much needed in the neighbourhood, and Dr. M'Kelvie's generosity is thoroughly appreciated.

The late Lady Jane Dundas has bequeathed £1,000 each to St. George's and King's College Hospitals, the Longmore Hospital for Incurables and the Convalescent Hospital at Edinburgh, and £5,000 to the Edinburgh Royal Infirmary. Middlesex and the London Fever Hospital are each to have £500; and she bequeaths £200 each to the Earlswood Asylum for Idiots, the Metropolitan Convalescent Institution, the Royal Hospital for Incurables, and the Belgrave Hospital for Children.

Dr. Frederick St. George Mivart has been appointed Medical Inspector of the Local Government Board, in place of the late Dr. Barry.

A meeting of the Special Appeal Committee of Charing Cross Hospital will be held at the Mansion House on Wednesday, November 3rd, at four p.m.

The foundation-stone of a new Union Hospital has been laid at Halifax by the Chairman of the Board of Guardians. The hospital will be entirely distinct from the workhouse, and is intended to accommodate 640 beds, at a cost of £130,000 for the erection.

The Committee of Management of Queen Charlotte's Lying-in Hospital, Marylebone Road, have received a donation of £1,000 from Mr. Henry L. Raphael, to name a bed in perpetuity in memory of the late Mrs. H. L. Raphael.

The meeting of the Council of the Metropolitan Hospital Sunday Fund on Wednesday was one of the most influentially attended and important which has ever been held in the history of this Fund. Dr. Glover has kept the out-patient question under the notice of the Council with commendable persistence during the last few years, and we congratulate the Distribution Committee and Dr. Glover upon the unanimity with which the important decisions come to were arrived at.

Maidstone's terrible experience has caused most sanitary authorities to exercise special watchfulness, and at Lynn an inquiry from the Local Government Board has elicited the fact that 120 cases of typhoid are present in the town, due, it is reported, to the exceptionally large quantity of surface water passing over pasture lands into the open stream from which the water of the town is drawn. The waterworks are held to blame. At Belfast a still more serious outbreak is reported, the number of patients amounting to upwards of 354.

The disinfection of the mains of the Maidstone Water Company was completed last week, those dealt with being all in connection with the Barming reservoir. The process consisted in first closing the sluices of the reservoir and emptying all the mains leading from it by opening hydrants in the lower parts of the town. Then the hydrants were closed, and a large quantity of bleaching lime having been put in the reservoir and thoroughly mixed with water by means of jets from a powerful Merryweather steam fire engine, the sluices were opened and the mixture admitted to the pipes. The fire engine was kept pumping into the reservoir for some hours, and jets of the disinfectant were used to thoroughly wash down the reservoir walls, so as to ensure every part being covered. The disinfectant was allowed to remain in the pipes during Sunday, and on Monday morning was allowed to escape, and the fire-engine pumped the reservoir dry. The Farley engines were then started, and the mains filled with fresh water, and it is now considered by the experts that all danger of infection from the typhoid bacillus in the pipes is at an end. The fire engine was employed to wash out the water-pipes and thoroughly flush the sewers at Barming Heath Asylum at the end of the week.

